# The Importance of Multidimensional Frailty in Clinical Practice

**DOI:** 10.3390/jcm13247645

**Published:** 2024-12-16

**Authors:** Nicola Veronese, Alberto Pilotto

**Affiliations:** 1 Faculty of Medicine, Saint Camillus International University of Health Sciences, 00161 Rome, Italy; 2 Department of Geriatric Care, Neurology and Rehabilitation, E.O. Galliera Hospital, Mura delle Cappuccine 14, 16128 Genoa, Italy; alberto.pilotto@galliera.it; 3 Department of Interdisciplinary Medicine, University of Bari “Aldo Moro”, 70121 Bari, Italy

Frailty in older adults is a complex, multidimensional syndrome characterized by decreased physiological reserves and increased vulnerability to stressors [1,2]. Traditionally, frailty assessments have focused primarily on two common models of frailty, i.e., the phenotypic [3] and accumulation of deficits models [4,5]. In brief, the first model focuses mainly on a muscle impairment that accelerates the transit from sarcopenia to frailty, and the second model is seen as an accumulation of deficits (yes/no) that finally lead to frailty. However, it is important to explain to readers the concept of multidimensional frailty [6] that better incorporates the notion of mutual interaction among physical, functional, psychological, and social factors [6,7], strongly related to the prognosis of older subjects [8,9]. For this reason, assessing multidimensional frailty is becoming increasingly crucial in clinical decision-making [10,11,12].

Traditional models of frailty are limited in daily clinical practice due to their narrow focus and complexity in real-world application [10]. They often fail to account for the heterogeneous nature of frailty, potentially underestimating risk or inappropriate interventions [13]. In contrast, multidimensional frailty models derived via Comprehensive Geriatric Assessment (CGA) may offer more holistic and realistic evaluation methods [14].

Since the use of CGA to detect multidimensional frailty is still largely unexplored, despite the importance of these topics in geriatric medicine, we launched this Special Issue entitled “Multidimensional Frailty: The Role of Comprehensive Geriatric Assessment”. In this Special Issue, we have collected several important contributions from all the world about multidimensional frailty and CGA.

One article validates the SELFY-BRIEF-MPI, a shortened, self-administered version of the Multidimensional Prognostic Index (MPI) for older adults. The study demonstrates a strong association between the SELFY-BRIEF-MPI and the standard MPI, indicating its potential as a reliable tool for frailty screening [15]. Involving 105 participants aged 65 and older, the study shows the tool’s accuracy in identifying multidimensional frailty, suggesting that it could facilitate early detection and intervention in various healthcare settings, such as the emergency department [16] and primary care, by simplifying the assessment of older adults at risk of frailty and adverse outcomes.

Another important contribution discusses the application of CGA for older adults in Sub-Saharan Africa, where healthcare resources and geriatrics training are limited [17]. The study highlights the challenges posed by an aging population and the increasing prevalence of non-communicable diseases in the region. While invasive treatments are growing in availability, no prior studies have assessed a CGA’s efficacy in managing the complex care needs of elderly patients requiring surgery or cancer treatment. The authors advocate for conducting research to adapt CGA to the region’s unique constraints, potentially improving elderly care and health outcomes [17].

Moreover, another study evaluates frailty and quality of life (QoL) in older patients with recurrent or metastatic head and neck squamous cell carcinoma (r/m HNSCC). CGA of 21 patients revealed significant frailty and impaired QoL, particularly regarding pain, fatigue, and oral function [18]. Psychological factors and prior HNSCC treatments contributed to reduced QoL. The study underscores the importance of integrating CGA into oncology to identify the unique needs of frail cancer patients and improve care through tailored multidisciplinary support [19], including prosthetic rehabilitation for improved oral function.

Furthermore, the group of Prof. Polidori contributes two important works our Special Issue. One study analyses geriatric syndromes (GSs) and resources in older patients with atrial fibrillation (AF), highlighting the challenges of managing AF with accompanying geriatric conditions. Using a CGA, it identifies higher multidimensional frailty in AF patients compared to non-AF peers, with specific deficits in sensory and cognitive functions. AF patients on oral anticoagulants exhibited mixed outcomes in resources and GSs, such as reduced age-appropriate living conditions but increased emotional resources. The findings emphasize the importance of using tailored management strategies for elderly AF patients, taking their complex health profiles into account to improve outcomes [20]. Another observational study of this group reported the importance of sarcopenia as a determinant of poor QoL after taking into account MPI, underlining the importance of early recognition of sarcopenia in hospitalized patients [21].

Finally, another study examines the effects of a 30-week multicomponent exercise program on frailty among community-dwelling older adults in Spain. Conducted with 360 participants, the program included aerobic, strength, and flexibility exercises. The results indicate reductions in frailty and pre-frailty, especially among older women in urban areas. Variability in outcomes was noted across different assessment tools, but the improvements persisted even after a training hiatus. The findings support using exercise programs as an effective intervention for reducing frailty, contributing to enhanced independence and quality of life among older populations [22].

We believe that our Special Issue could be of importance for several reasons. Embracing a multidimensional approach to frailty assessment, exemplified by the CGA, is crucial for accurately identifying and managing frailty in older adults [23]. By considering the interplay of the domains assessed by CGA, such as physical, cognitive, psychological, and social factors, healthcare providers can develop personalized interventions that improve patient outcomes and quality of life. Ongoing research and innovation in this field will further refine assessment tools and care strategies, ultimately enhancing the well-being of the aging population.
